# Central nervous system transcriptome of *Biomphalaria alexandrina*, an intermediate host for schistosomiasis

**DOI:** 10.1186/s13104-017-3018-6

**Published:** 2017-12-11

**Authors:** Tamer A. Mansour, Mohamed R. Habib, Laura C. Vicente Rodríguez, Anthony Hernández Vázquez, Julián Maldonado Alers, Alfredo Ghezzi, Roger P. Croll, C. Titus Brown, Mark W. Miller

**Affiliations:** 1 Department of Population Health and Reproduction, University of California, Davis, CA USA; 2 Department of Clinical Pathology, College of Medicine, Mansoura University, Mansoura, Egypt; 3 Medical Malacology Department, Theodor Bilharz Research Institute, Giza, 12411 Egypt; 4 Institute of Neurobiology and Department of Anatomy & Neurobiology, University of Puerto Rico, Medical Sciences Campus, 201 Blvd del Valle, San Juan, Puerto Rico; 5 Department of Biology, University of Puerto Rico, Río Piedras Campus, San Juan, Puerto Rico; 6 Department of Physiology and Biophysics, Dalhousie University, Halifax, NS Canada

**Keywords:** *Biomphalaria alexandrina*, *Schistosoma mansoni*, CNS, Trematode, Gastropod, Mollusk, Nile

## Abstract

**Objective:**

Globally, more than 200 million people live at risk of the neglected tropical disease schistosomiasis (or snail fever). Larval schistosomes require the presence of specific snail species that act as intermediate hosts, supporting their multiplication and transformation into forms that can infect humans. This project was designed to generate a transcriptome from the central nervous system (CNS) of *Biomphalaria* *alexandrina*, the major intermediate host for *Schistosoma mansoni* in Egypt.

**Results:**

A transcriptome was generated from five pooled central nervous systems dissected from uninfected specimens of *B.* *alexandrina*. Raw Illumina RNA-seq data (~ 20.3 million paired end reads of 150 base pairs length each) generated a transcriptome consisting of 144,213 transcript elements with an N50 contig size of 716 base pairs. Orthologs of 15,246 transcripts and homologs for an additional 16,810 transcripts were identified in the UniProtKB/Swiss-Prot database. The *B. alexandrina* CNS transcriptome provides a resource for future research exploring parasite-host interactions in a simpler nervous system. Moreover, increased understanding of the neural signaling mechanisms involved in the response of *B.* *alexandrina* to infection by *S.* *mansoni* larvae could lead to novel and highly specific strategies for the control of snail populations.

**Electronic supplementary material:**

The online version of this article (10.1186/s13104-017-3018-6) contains supplementary material, which is available to authorized users.

## Introduction

Schistosomiasis remains one of the most prevalent neglected tropical diseases affecting human populations in many parts of Africa, Asia, and South America. The World Health Organization (WHO) estimated that more than 218 million people in 78 countries required preventive chemotherapy in 2015 [1]. The WHO has recommended a multifaceted strategic plan for control and prevention of schistosomiasis, including large-scale chemotherapy for high-risk populations, hygiene education, access to safe drinking water, and snail control [2].

Fresh water pulmonate snails from the genus *Biomphalaria* act as the obligatory intermediate host for *Schistosoma mansoni*, the trematode species that causes intestinal schistosomiasis. A recent whole genome analysis for *Biomphalaria glabrata*, the major intermediate host in the Western Hemisphere, identified several potential targets for developing novel control measures [3]. In Egypt, where schistosomiasis dates to antiquity [4–6], *Biomphalaria alexandrina* is the predominant intermediate host for *S.* *mansoni* [7–9]. The presence of *B.* *alexandrina* is a key factor that determines the prevalence of intestinal schistosomiasis in the country.

In 2016, the Ministry of Health and Population of Egypt (MoHP) and WHO conducted a mapping of *S.* *mansoni* infection in five Nile Delta governorates [10]. The results of this project showed that prevalence rates ranged from 4.7% in Qalyubia Governorate to 17.6% in Sharqia Governorate, with an average prevalence of 10.7% in the five governorates surveyed. These observations indicate that previous assessments may have underestimated the extent of infection.

Efforts to control snail populations, such as molluscicides or introduction of predator species, have yielded only modest results. One potential target to be considered for snail control is its central nervous system (CNS), since it regulates vital functions including cardiac activity, feeding and reproductive behavior. In the present investigation, a neural transcriptome was generated with the ultimate goal of identifying signaling mechanisms involved in the response of *B.* *alexandrina* to infection by *S.* *mansoni* larvae. Such mechanisms may lead to novel and highly specific strategies for the control of snail populations.

## Main text

### Methods

#### Sample collection and preparation

**Fig. 1:**
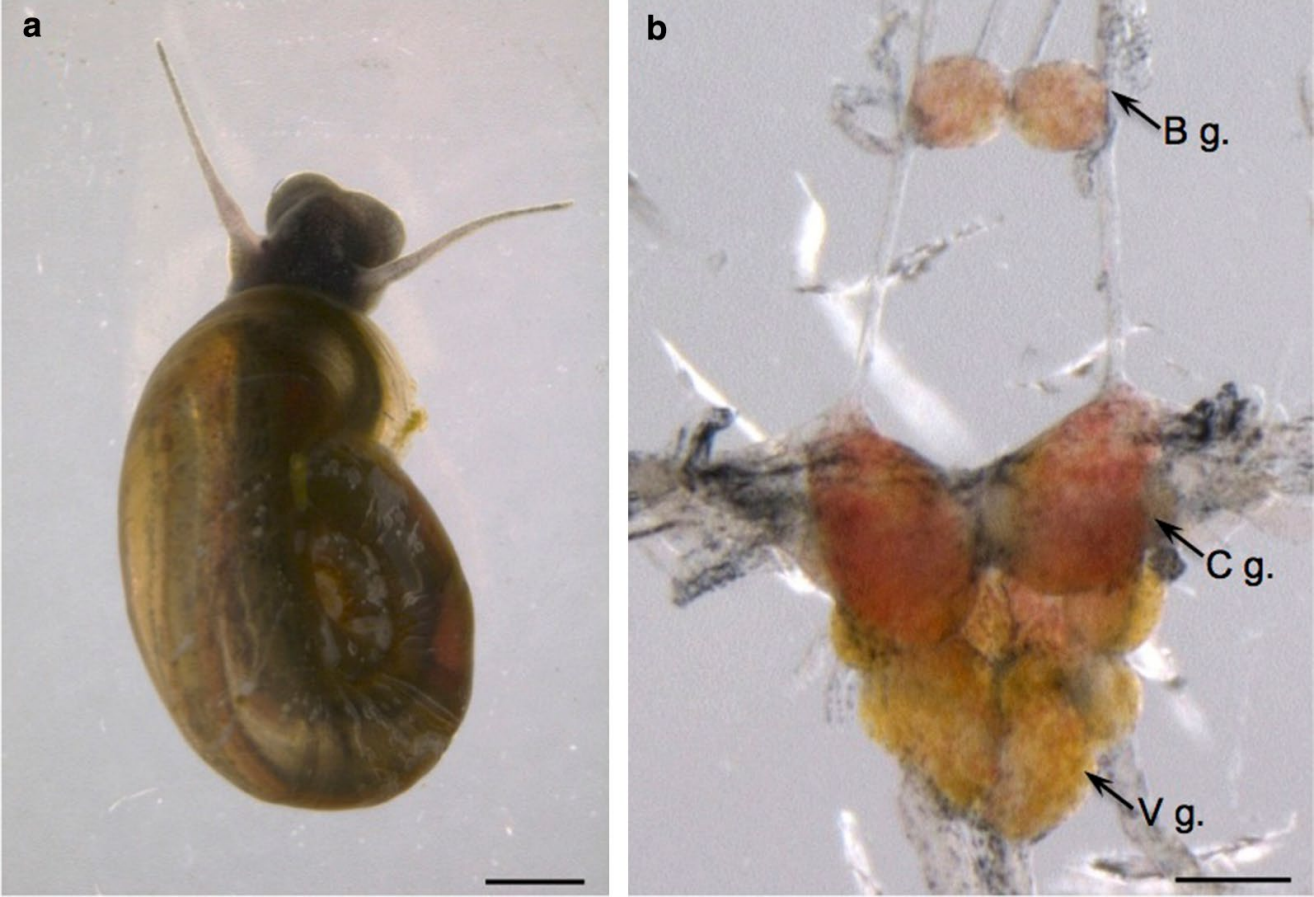
Source of RNA used to generate transcriptome. **a** *Biomphalaria alexandrina* specimen. Calibration bar: 1 mm. **b** Dissected central nervous system. *B g.*, buccal ganglion; *C g.*, cerebral ganglion; *V* *g.*, visceral ganglion. Calibration bar: 200 μm

Specimens of *B.* *alexandrina* (Fig. 1a) used in the present study were descendants from a field population that was collected from Giza Governorate in Egypt in 2012 and shipped to the Faculty of Medicine, Dalhousie University, Canada. They were maintained under a 14:10 light–dark cycle and fed romaine lettuce ad libitum. The central nervous systems (Fig. 1b) were dissected, immediately submerged in RNAlater (Thermo Fisher Scientific, USA), and stored at 4 °C for further analysis. Five pooled nervous systems were homogenized in lysis-binding solution provided in the RNAqueous-Micro Total RNA Isolation Kit (Thermo Fisher Scientific, USA). Total RNA was isolated following the manufacturer’s instructions. The RNA was quantified using a NanoDrop spectrophotometer and its quality was verified with an agarose gel. Samples were then sent to the Genomic Sequencing and Analysis Facility (GSAF) at the University of Texas at Austin for library preparation and Illumina sequencing. PolyA RNA selection was implemented using the Poly(A)Purist MAG Kit (Life Technologies). The mRNA quality was assessed with an Agilent Bioanalyzer. A non-directional RNA-seq library (cDNA inserts of approximately 384 bp) was generated using NEBNext Module Components. Sequencing was performed on an Illumina HiSeq 4000 sequencer (Paired End 2 × 150 bp).

#### Transcriptome preprocessing, assembly and annotation

**Fig. 2:**
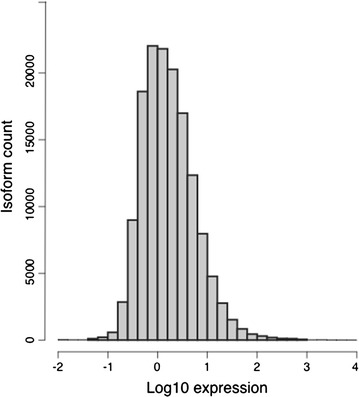
Histogram of log TPM expression

Quality based trimming was implemented using Trimmomatic v0.33 [11] followed by K-mer spectral analysis to remove low abundance K-mers using the Khmer 2.0 package [12]. FastQC v0.11.3 was used to check data quality before and after trimming [13]. Filtration of input sequences produced ~ 14.4 million paired end (PE) reads and ~ 5.4 million single end (SE) reads. High-quality fragments were pooled for de novo transcriptome assembly using Trinity v2.2.0 [14] producing 149,545 transcripts. Several filtration procedures were performed to clean up the assembly: SeqClean was used to trim poly-A tails and remove 38 low complexity sequences [15]. Custom scripts were used to remove 745 fragments smaller than 200 bp. Back-mapping of input sequencing reads to the assembly using Salmon software, allowed removal of 4526 uncovered isoforms [16]. Finally, scanning the new assembly against the UniVec Database containing vector and artificial sequences eliminated 23 transcripts and trimmed an additional 98 transcripts [17]. In total, all filtration steps excluded 5332 transcripts. The final assembly was composed of 144,213 isoforms that belong to 128,739 genes. The total assembly size was 82.7 Mb with N50 equal to 716 base pairs. (Assembly statistics: Table 1). To evaluate the quality of the final assembly, another round of back mapping of input sequencing reads to the assembly was performed. PE and SE reads showed mapping rates of about 96 and 89% respectively. For benchmarking, Universal Single-Copy Orthologs (BUSCO v.2) software was used to assess annotation completeness against single-copy orthologs in Metazoa (978 orthologs) [18, 19]. BUSCO analysis was able to identify 79.5, 19.5, 1.1% complete, fragmented, and missing gene models. To assess expression abundance of assembled transcripts, mapped sequence reads were normalized into transcript per million scale (TPM) and logarithmic transformation of the normalized expression was plotted as a histogram (Fig. 2). Most of the transcripts had expression abundance levels around 1.5 TPM but only 848 transcripts had TPM values greater than 100 (Expression statistics: Table 2).

**Table 1:** Transcriptome assembly statistics

Total trinity ‘genes’	128739
Total trinity transcripts	144213
Maximum length	5634
Minimum length	201
Percent GC	36.75
Statistics based on ALL transcript contigs	
Contig N10	1986
Contig N20	1459
Contig N30	1128
Contig N40	896
Contig N50	716
Median contig length	402
Mean contig length	573
Total assembled bases	82,672,833
Statistics based on LONGEST ISOFORM per “GENE”	
Contig N10	1797
Contig N20	1298
Contig N30	1001
Contig N40	796
Contig N50	636
Median contig length	382
Mean contig length	533
Total assembled bases	68,601,167

**Table 2:** Expression abundance statistics

Mean expression	6.93
Median expression	1.47
Maximum expression	27609
Isoforms with exp > 10 TPM	11187
Isoforms with exp > 100 TPM	848
Isoforms with exp > 1000 TPM	85
Isoforms with exp > 10000 TPM	3

For annotation, TransDecoder v2.0.1 was used to predict open reading frames (ORFs) [20]. A reciprocal BLAST search was implemented between the final assembly and target databases using BLAST plus v2.2.30. Significant BLAST hits (E-values < 10e−5) were utilized by crb-BLAST software to identify orthologous transcripts [21]. Transcripts with significant BLAST hits that failed to find a significant ortholog were annotated by the best BLAST hit as a candidate homolog.

### Results

Annotation of all transcripts in the final assembly predicted 40,408 transcripts with long open reading frames (≥100 amino acids) (Additional file 1). Orthologs of 15,246 transcripts were identified by conditional BLAST search against the UniProtKB/Swiss-Prot database (Additional file 2). Homologs for an additional 16,810 transcripts were identified by the best BLAST hit against the same database (Additional file 3).

Each transcript in the FASTA file of the transcriptome was annotated with its transcript length and expression level in TPM values. Also, if identified, transcripts were annotated by their orthologs. Otherwise, annotation was implemented using homologs or with possible ORFs (Additional file 4).

Of the 848 highly expressed transcripts, approximately 49% corresponded to orthologs in the Swiss-Prot database. An additional 11% had homologs from a BLAST search against the same database. Another 17% had long ORFs, > 60% of which were complete, but failed to attain significant BLAST hit against the Swiss-Prot database. (Annotation Statistics: Table 3).

**Table 3:** Annotation statistics

	Total transcriptome (144,213 transcripts)		Highly expressed (848 transcripts)	
	Count	Percent	Count	Percent
Transcripts with long ORF	40408	28.02	336	39.62
Transcripts with complete long ORF	8678	6.02	258	30.42
Against Swiss-Prot database				
Significant BLAST match	32056	22.23	428	50.47
Transcripts with orthologs	15246	10.57	336	39.62
Transcripts with homologs	16810	11.66	92	10.85
Transcripts with ORF but no BLAST hit	15161	10.51	145	17.10
Against (nr) BLAST database				
Significant BLAST match	–	–	542	63.92
Transcripts with orthologs	–	–	469	55.31
Transcripts with homologs	–	–	73	8.61
Transcripts with ORF but no BLAST hit	–	–	61	7.19

The 848 highly expressed transcripts were selected for a second reciprocal BLAST search against the comprehensive NCBI Non-Redundant (NR) BLAST database. About 55% of transcripts were assigned an ortholog and 9% were annotated with a best homolog. Only 7% of the highly expressed transcripts had long ORF but failed to achieve a significant hit against the NR database. A new FASTA file for the highly expressed transcripts was annotated by the BLAST results against the NR database (Additional file 5). A reciprocal BLAST search against the recently published transcriptome of twelve pooled *B. glabrata* tissues [3] showed 22,613 orthologs with average identity 99.1% (Additional file 6).

### Discussion

The relatively simple nervous systems of gastropod mollusks contain large identified neurons that allow detailed electrophysiological, biochemical, and molecular analyses at the cellular level [22–24]. Gastropods therefore serve as promising models for neurobiological studies exploring the cellular basis of behavior, including sensorimotor integration [25, 26], central pattern generator (CPG) networks [27, 28], neuroendocrine regulation of reproduction [29, 30], and responses to parasitism [31, 32]. The transcriptome of the *B. alexandrina* CNS complements the whole genome characterization of *B. glabrata* [3], and provides a resource for future investigation of parasite-host interactions with biomedical implications in a highly tractable nervous system. This transcriptome should also lead to novel strategies directed toward snail control.

## Limitations

Potential limitations may include:

1. Short read sequencing can produce an incomplete gene model leading to inaccurate identification of multiple isoforms for the same gene.
2. RNA preparation with Poly(A) selection allows us to enrich for non-ribosomal transcripts but also will result in loss of non-polyadenylated transcripts.
3. Lack of biological and technical replicates.

## Additional files

**Additional file 1.** Coding Transcripts: Transcripts with long open reading frames (≥100 amino acids).    

**Additional file 2.** Orthologous Transcripts: Transcripts with significant mutual BLAST hits against the UniProtKB/Swiss-Prot database.

**Additional file 3.** Homologous Transcripts: Transcripts with significant one-way BLAST hits against the UniProtKB/Swiss-Prot database.

**Additional file 4.** Final assembly: Transcripts annotated with transcript length and expression level in TPM values. Also, if identified, transcripts were annotated by their orthologs. Otherwise, annotation was implemented using homologs or with possible open reading frames.

**Additional file 5.** Hi-Express transcripts: Highly expressed transcripts annotated by the BLAST results against the comprehensive NCBI Non-Redundant (NR) database.

**Additional file 6.** BLAST against *B. glabrata*: A reciprocal BLAST search against the recently published transcriptome of twelve pooled *Biomphalaria glabrata* tissues [3].

## Abbreviations

BLAST: Basic Local Alignment Search Tool
NCBI: National Center for Biotechnology Information
ORF: open reading frame
WHO: World Health Organization
*B g.*: buccal ganglion
*C g.*: cerebral ganglion
*V g.*: visceral ganglion

## References

[1] WHO 2011. Report of an informal consultation on schistosomiasis control. Geneva, Switzerland. (WHO/HTM/NTD/PCT/2013.3). http://www.who.int/neglected_diseases/resources/9789241505017/en/. Accessed 29 May 2017.

[2] WHO 2017. Schistosomiasis Fact Sheet N115. http://www.who.int/mediacentre/factsheets/fs115/en/. 2017. Accessed 29 May 2017.

[3] Adema CM, Hillier LW, Jones CS, Loker ES, Knight M (2017). Whole genome analysis of a schistosomiasis-transmitting freshwater snail. Nat Commun..

[4] Ruffer M (1910). Note on the presence of ‘‘Bilharzia haematobia’’ in Egyptian mummies of the twentieth dynasty [1250-1000 B.C.]. Br Med J.

[5] Contis G, David A (1996). The epidemiology of Bilharzia in ancient Egypt: 5000 years of schistosomiasis. Parasitol Today.

[6] Barakat RM (2013). Epidemiology of schistosomiasis in Egypt: travel through time: review. J Adv Res..

[7] Lotfy WM, Dejong RJ, Abdel-Kader A, Loker ES (2005). A molecular survey of *Biomphalaria* in Egypt: is *B. glabrata* present?. Am J Trop Med Hyg.

[8] Lotfy WM, Dejong RJ, Black BS, Loker ES (2005). Specific identification of Egyptian *Biomphalaria* species and possible hybrids using the polymerase chain reaction based on nuclear and mitochondrial loci. Mol Cell Probes.

[9] Abou-El-Naga IF (2013). *Biomphalaria alexandrina* in Egypt: past, present, and future. J Biosci.

[10] Haggag AA, Rabiee A, Abd Elaziz KM, Gabrielli AF, Abdel Hay R, Ramzy RM (2017). Mapping of *Schistosoma mansoni* in the Nile Delta, Egypt: assessment of the prevalence by the circulating cathodic antigen urine assay. Acta Trop.

[11] Bolger AM, Lohse M, Usadel B (2014). Trimmomatic: a flexible trimmer for Illumina sequence data. Bioinformatics.

[12] Crusoe MR, Alameldin HF, Awad S, Boucher E, Caldwell A, Cartwright R (2015). The khmer software package: enabling efficient nucleotide sequence analysis. F1000Res.

[13] Andrews S. FastQC: a quality control tool for high throughput sequence data. 2010. http://www.bioinformatics.babraham.ac.uk/projects/fastqc/. Accessed 29 May 2017.

[14] Grabherr MG, Haas BJ, Yassour M, Levin JZ, Thompson DA, Amit I (2011). Full-length transcriptome assembly from RNA-Seq data without a reference genome. Nat Biotechnol.

[15] Masoudi-Nejad A, Tonomura K, Kawashima S, Moriya Y, Suzuki M, Itoh M, Kanehisa M, Endo T, Goto S (2006). EGassembler: online bioinformatics service for large-scale processing, clustering and assembling ESTs and genomic DNA fragments. Nucleic Acids Res.

[16] Patro R, Duggal G, Love MI, Irizarry RA, Kingsford C (2017). Salmon provides fast, and bias-aware quantification of transcript expression. Nat Methods.

[17] The UniVec Database. https://www.ncbi.nlm.nih.gov/tools/vecscreen/univec/. Accessed 29 May 2017.

[18] Simão FA, Waterhouse RM, Ioannidis P, Kriventseva EV, Zdobnov EM (2015). BUSCO: assessing genome assembly and annotation completeness with single-copy orthologs. Bioinformatics.

[19] Creevey CJ, Muller J, Doerks T, Thompson JD, Arendt D, Bork P (2011). Identifying single copy orthologs in Metazoa. PLoS Comput Biol.

[20] TransDecoder. http://transdecoder.github.io. Accessed 29 May 2017.

[21] Aubry S, Kelly S, Kumpers BM, Smith-Unna RD, Hibberd JM (2014). Deep evolutionary comparison of gene expression identifies parallel recruitment of trans-factors in two independent origins of C4 photosynthesis. PLoS Genet.

[22] Kandel ER (1976). Cellular basis of behavior: an introduction to behavioral neurobiology.

[23] Kandel ER (1979). Behavioral biology of *Aplysia*.

[24] Chase RM (2002). Behavior and its neural control in gastropod molluscs.

[25] Levi R, Varona P, Arshavsky YI, Rabinovich MI, Selverston AI (2005). The role of sensory network dynamics in generating a motor program. J Neurosci.

[26] Latorre R, Levi R, Varona P (2013). Transformation of context-dependent sensory dynamics into motor behavior. PLoS Comput Biol.

[27] Getting PA (1989). Emerging principles governing the operation of neural networks. Annu Rev Neurosci.

[28] Katz PS, Frost WN (1996). Intrinsic neuromodulation: altering neuronal circuits from within. Trends Neurosci.

[29] Lever J, Boer HH, eds. Molluscan neuro-endocrinology. Amsterdam: North-Holland Publishing Company; 1983.

[30] Koene JM (2010). Neuro-endocrine control of reproduction in hermaphroditic freshwater snails: mechanisms and evolution. Front Behav Neurosci.

[31] de Jong-Brink M (1995). How schistosomes profit from the stress responses they elicit in their hosts. Adv Parasitol.

[32] Hoek RM, van Kesteren RE, Smit AB, de Jong-Brink M, Geraerts WPM (1997). Schistosome parasites directly induce changes in gene expression in the central nervous system of their molluscan host. Proc Natl Acad Sci USA.

